# Estimating the economic burden of respiratory syncytial virus infection among children <2 years old receiving care in Maputo, Mozambique

**DOI:** 10.7189/jogh.15.04076

**Published:** 2025-04-11

**Authors:** Neele Rave, Tufária Mussá, An Nguyen, Clint Pecenka, Farina L Shaaban, Louis J Bont, RSV GOLD III – Health Economics Study Group, Fadlulai Abdu-Raheem, Anas Abubakar, Rosemary Akuaku, Abdullahi Aminu, Louis Bont, Ram H Chapagain, Assucênio Chissaque, Andrew Clark, Joycelyn Dame, Frédéric Debellut, Nilsa de Deus, Rita Dhital, Upendra Dhungana, Amma Ekem, Norbert Fuhngwa, Maria A Garba, Fatima J Giwa, Bamenla Goka, Esperança L Guimarães, Prakash Joshi, Ranju Karki, Habiba Lawal, Bernsah D Lawong, Braiton Maculuve, Henshaw Mandi, Elias Manjate, Yara Manjate, Izilda Matimbe, Abdullahi Musa, Tufária Mussá, Teddy Naddumba, Adita Nepali, An Nguyen, Ebenezer Ntow, Aira A Olorukooba, Kwabena A Osman, Mirela Pale, Cesar Palha, Uttam Paudel, Clint Pecenka, Neele Rave, Farina L Shaaban, Arun K Sharma, Rupesh Shrestha, Cristina Sinussene, Nirasta Thakili, Farida Zavala

**Affiliations:** 1 Department of Paediatrics, University Medical Centre Utrecht, Utrecht, The Netherlands; 2 Faculty of Medicine, University Eduardo Mondlane, Maputo, Mozambique; 3 Center for Vaccine Innovation and Access, PATH, Ho Chi Minh City, Vietnam; 4 Center for Vaccine Innovation and Access, PATH, Seattle, Washington, USA

## Abstract

**Background:**

Data on costs of respiratory syncytial virus (RSV) in low- and lower-middle-income countries are urgently needed to inform the introduction of recently developed vaccines. We estimated the costs of lower respiratory tract infections associated with RSV infection in Mozambique.

**Methods:**

We conducted a prospective cohort study to assess household and societal costs of RSV infection in children <2 years old who sought care in a referral hospital or a primary health centre in Mozambique during one local RSV season (February to August 2023). We used molecular point-of-care testing to confirm RSV status. We collected direct medical and non-medical costs and indirect cost data from hospital records and patient-level questionnaires at the initial visit and 2–4 weeks post-discharge.

**Results:**

We recruited 544 children; 52.6% were girls and the median age was 9.3 months. From the sample, 286 children from the outpatient department, 233 from the paediatric wards, and 25 from the intensive care unit (ICU). RSV was confirmed in 42 (14.7%) outpatients, 111 (47.6%) inpatients, and 6 (24.0%) ICU cases. The mean total costs associated with RSV were USD 43 (95% confidence interval (CI) = 11–76) for outpatients, USD 612 (95% CI = 544–680) for inpatients, and USD 1161 (95% CI = 837–1485) for ICU cases. The government covered 16.9%, 89.9%, and 80.0% of overall societal costs for outpatients, inpatients, and ICU patients, respectively. The average household out-of-pocket costs for life-threatened RSV cases were more than 1.5 times the monthly minimum wage of USD 91, causing a high financial burden on families in Mozambique.

**Conclusions:**

RSV infection represents a significant healthcare and economic burden in children <2 years old. Our results provide input for cost-effectiveness analyses and informed decision-making when considering RSV immunisation in Mozambique.

Respiratory syncytial virus (RSV) causes a substantial burden of acute lower respiratory tract infections (LRTIs) in children under five years old, with approximately 33 million RSV-associated respiratory tract infections and 118 000 deaths estimated to occur annually in this group [1]. Most infections (95%), as well as more than 97% of deaths related to RSV, occur in low- and middle-income countries (LMICs) [2].

Targeted intervention strategies could significantly influence RSV-associated respiratory disease outcomes. While several preventive interventions are still under development [3], two have recently been approved. One of them, nirsevimab – a long-acting monoclonal antibody (mAb) given as a single injection to infants for coverage throughout the whole RSV season – demonstrated significant benefits in a multinational randomised controlled trial [4]. The second intervention involved a phase III maternal immunisation trial, which showed promising results for a bivalent prefusion F vaccine administered to pregnant women during the third trimester, which effectively protects infants against RSV for up to six months postpartum [5].

RSV-related health burden and cost data in LMICs, specifically in Mozambique, are scarce. In Mozambique, approximately 24.8% of all childhood deaths are attributed to LRTIs [6], with RSV playing a substantial role [7]. A hospital-based study conducted in Maputo, Mozambique, between 2017 and 2018, showed an RSV infection incidence rate of 23.1% among children <2 years old [8]. Another study estimated RSV infection incidence rates of 58.3 (95% confidence interval (CI) = 43.0–79.0) per 1000 children [2]. A more recent study on the intensive care and high-dependency unit in Maputo reported that 35.1% of patients admitted with severe acute respiratory infection symptoms tested positive for RSV [9].

As prevention strategies emerge, determining the burden of RSV and the associated costs of care in LMICs is critical and will improve our understanding of the economic and healthcare burden that may be averted by the introduction of those strategies. This information will assist stakeholders in prioritising and guiding evidence-based selection among available preventive interventions, particularly in contexts where resources are scarce. Therefore, we aimed to determine the direct medical and non-medical costs as well as indirect costs of LRTIs associated with RSV infection among children seeking care in urban healthcare facilities in Maputo, Mozambique, from the perspectives of the health system, households, and society.

## METHODS

### Study setting

We conducted this prospective study as part of the multi-country RSV Global Online Mortality Database (GOLD) III – Health Economics Study, which took place in Ghana, Mozambique, Nepal, and Nigeria. All four countries were receiving financial and programmatic support from Gavi, the Vaccine Alliance. The study took place during one local RSV season and built upon the RSV GOLD III – Intensive Care Unit (ICU) Network Study, which was previously conducted in these countries to investigate the burden of life-threatening RSV [9]. In Mozambique, the study was conducted at Maputo Central Hospital referral hospital and Maputo’s 1st of May Health Centre primary healthcare centre.

A national policy emphasising primary healthcare, equity, and quality services guides the health system in Mozambique. International aid is a significant source of financing; it prioritises initiatives targeting major health issues such as HIV/AIDS, malaria, tuberculosis, and maternal as well as child health. Urban areas have seen diversification in health financing, although this remains a small portion of overall funding. In the two health facilities covered in our study, donations from international aid also play a large role in financing medications, medical supplies, and facility equipment. We consider costs for patient care requiring such resources a health system cost.

### Study population

All children <2 years old who visited either of the healthcare facilities with (severe) acute respiratory infection during the local RSV season, *i.e.* between February and August 2023, were eligible for inclusion. We excluded children aged below 28 days and those with an onset of symptoms occurring more than ten days before the facility visit.

We categorised cases by severity: non-severe (outpatient only), severe (inpatient, no ICU admission), and life-threatened (ICU admission or fatality). Study staff were available during weekdays. Children admitted during the weekend were screened via admission books and clinical files on Mondays and, if eligible, were included in the study. At the 1st of May Health Centre, no children were recruited during the weekend. A nasopharyngeal swab was obtained within 72 hours and tested for RSV using the molecular point-of-care ID Now RSV test [10,11]; we included both RSV-positive and RSV-negative patients in our analysis, allowing for a comparison of costs for RSV and non-RSV cases.

The National Committee for Bioethics in Health (Ref: 806/CNBS/22) and the Medical Research Ethics Committee NedMec (#20-536) of the University Medical Centre in Utrecht, the Netherlands, approved the study protocol.

### Sample size calculations

To determine the sample size needed for estimating the costs of LRTIs associated with RSV infection, we used the following formula [12]:



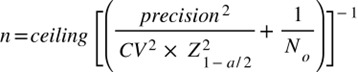



We used a Z-score of 1.96 and its 95% confidence interval (CI), a coefficient of variation of 0.5, and precision at ±10% of the mean cost. We used the caseload (N_0_) and RSV-positivity rate of 35.1% from a previous RSV study conducted at the ICU at Maputo Central Hospital [9] to calculate the target sample size. We estimated that, to achieve the optimal sample size of 91 RSV-positive inpatients and 94 RSV-positive outpatients, we had to enrol 260 inpatients and 309 outpatients with LRTI. Enrolment numbers may range from 122 to 910 LRTI inpatients and 128 to 900 LRTI outpatients, depending on the observed prevalence during the RSV season.

### Data collection

We collected the data during one local respiratory season, from February until August 2023. Trained study staff administered questionnaires designed for the RSV GOLD III – Health Economics Study, which were translated into Portuguese. Caregiver questions covered demographics and socioeconomic status, pre-facility visit illness history, and related costs. In addition, all costs from the day of the visit were reported. Patient medical records were reviewed upon discharge for detailed information on laboratory tests, imaging, procedures, and medication. For both inpatients and outpatients, follow-up phone calls occurred two to four weeks after facility visit/discharge to obtain information on subsequent illness-related costs, whether any additional facility visits took place for illness-related symptoms, and, if so, which costs were incurred. We assessed cost data from the health system and household perspectives, which were then combined to calculate the total costs from the societal perspective. A detailed overview of the study setup, the cost analysis (including the various cost components and the bottom-up approach used for data collection and analysis), can be found in the **Online Supplementary Document**. The local study team entered all data into the Castor electronic data capture system [13].

### Data analysis

We summarised the demographic and clinical characteristics of all patients. We used a bottom-up costing approach to estimate the treatment costs associated with respiratory illness. In general, we calculated direct medical and non-medical costs by multiplying the quantity used by unit cost for all items across all cost categories. For indirect costs, we considered the income loss reported by the household, as well as productivity loss. We determined total costs as the sum of direct medical costs, direct non-medical costs, and indirect costs. Furthermore, we analysed the costs concerning the severity of cases based on the location of admission.

### Statistical analysis

Since almost all cost data were non-normally distributed, we used both means and medians to summarise cost data. We checked for skewness and used bootstrapping with 1000 replications to obtain the 95% CI for the mean cost since the data were not normally distributed. The lower limits of the 95% CI were censored to zero to reflect the fact that cost data were nonnegative. We used the Mann-Whitney U test to compare the non-normally distributed continuous variables between RSV-positive and RSV-negative cases and compared costs for non-severe, severe, and life-threatened groups using the Kruskal-Wallis test, considering a *P*-value ≥0.05 to indicate no significant difference between the groups. We conducted all statistical analyses separately for RSV-positive and RSV-negative patients. The analyses were done using Stata, version 18.0 (StataCorp LLC, College Station, TX, USA).

## RESULTS

### Patient characteristics

**Figure 1:**
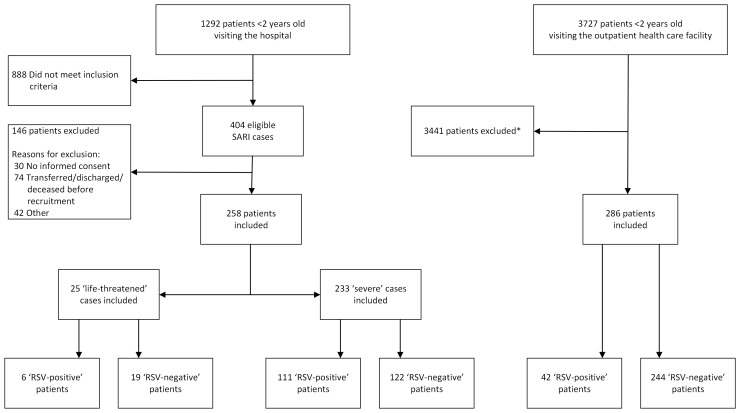
Flow diagram illustrating the total number of outpatients and inpatients included and excluded from the study. RSV – respiratory syncytial virus, SARI – severe acute respiratory infection. *Reasons for exclusion unknown, not included.

We included 544 patients in the study (**Figure 1**), of whom 286 (52.6%) attended the 1st of May Health Centre, 233 (42.8%) were admitted to the general wards, and 25 (4.6%) were admitted to the ICU. An overview of weekly counts of recruited children during the study period is provided in the supplementary materials (Figure S2 in the **Online Supplementary Document**). Almost a third (n = 159, 29.2%) of included children tested positive for RSV (**Table 1**). The mean age of RSV-positive inpatient cases was 6.9 months (standard deviation (SD) = 6.3) at the time of enrolment, and the majority were female (n = 89, 56.0%). The median length of stay among RSV-positive inpatients was 4.3 days (interquartile range (IQR) = 3–5) for severe cases and six days (IQR = 5–8) for life-threatened cases. Of all patients, 193 (35.5%) sought care before the index visit, more often (*P* = 0.0034) among those testing positive for RSV (n = 67, 44.4%) compared to non-RSV cases (n = 126, 32.1%).

**Table 1:** Demographics and clinical characteristics of RSV-positive and RSV-negative children <2 years old by severity level*

	RSV-positive				RSV-negative				All patients
	**Total**	**Non-severe**	**Severe**	**Life-threatened**	**Total**	**Non-severe**	**Severe**	**Life-threatened**	**Total**
**Total number of participants**	159	42	111	6	385	244	122	19	544
**Age in months, x̄ (SD)**	6.9 (6.3)	10.0 (7.1)	5.9 (5.8)	3.3 (2.1)	10.2 (6.4)	10.2 (6.4)	10.5 (6.4)	9.4 (7.0)	9.3 (6.6)
**Female**	89 (56.0)	26 (61.9)	58 (52.3)	5 (83.3)	197 (51.2)	126 (51.6)	61 (50.0)	10 (52.6)	286 (52.6)
**Household size, MD (IQR)**	5 (4–6)	6 (4–7)	5 (4–6)	4 (3–5)	5 (4–7)	5 (4–7)	5 (4–6)	5 (3–6)	5 (4–7)
**Level of education of primary caregiver secondary school or higher**	140 (88.1)	32 (76.2)	103 (92.8)	5 (83.3)	330 (85.7)	202 (82.8)	114 (93.4)	14 (73.7)	470 (86.4)
**Distance to healthcare facility in km, x̄ (SD)**	11.4 (9.9)	3.1 (2.3)	14.2 (9.0)	18.2 (21.5)	9.8 (23.1)	3.0 (3.8)	18.8 (31.6)	39.6 (48.4)	10.3 (20.1)
**Diagnosis at admission**									
Bronchiolitis	78 (50.7)	0 (0.0)	75 (70.1)	3 (60.0)†	41 (10.9)	0 (0.0)	39 (33.1)	2 (11.8)	119 (22.4)
Pneumonia	32 (20.8)	0 (0.0)	30 (28.0)	2 (40.0)†	77 (20.4)	0 (0.0)	72 (61.0)	5 (29.4)	109 (20.5)
URTI	39 (25.3)	39 (92.9)	0 (0.0)	0 (0.0)	223 (59.0)	221 (91.0)	2 (1.7)	0 (0.0)	262 (49.3)
Other	5 (3.3)	3 (7.1)	2 (1.9)	0 (0.0)	37 (9.8)	22 (9.1)	5 (4.2)	10 (58.8)	42 (7.9)
**Prematurity**	27 (17.0)	7 (16.7)	19 (17.1)	1 (16.7)	32 (8.4)	16 (6.6)	13 (10.8)	3 (16.7)	59 (11.0)
**Comorbidity**	18 (11.3)	2 (4.8)	15 (13.5)	1 (16.7)	45 (11.7)	20 (8.2)	21 (17.2)	4 (21.1)	63 (11.6)
**Length of stay in days, MD (IQR)**	4 (3–5)	N/A	4 (3–5)	6 (5–8)†	3 (2–5)	N/A	3 (2–4)	12 (6–21)^‡^	3 (2–5)
**Previous medical consultation**	71 (44.9)	12 (29.3)	53 (47.8)	6 (100)	122 (31.7)	52 (21.3)	53 (43.4)	17 (89.5)	193 (35.5)
**Follow-up care**	102 (68.9)	9 (24.3)^§^	89 (84.0)	4 (80.0)†	152 (47.1)	32 (16.7)^¶^	108 (91.5)	12 (92.3)^║^	254 (53.9)
**Mortality**	1 (0.7)	0 (0.0)	0 (0.0)	1 (16.7)	4 (1.1)	0 (0.0)	0 (0.0)	4 (23.5)††	5 (1.0)

IQR – interquartile range, MD – median, RSV – respiratory syncytial virus, SD – standard deviation, URTI – upper respiratory tract infection, x̄ – mean

*Values presented as n (%) unless specified otherwise.

†Missing data due to incomplete or missing clinical records (n = 5/6).

‡Missing data due to incomplete or missing clinical records (n = 15/19).

§Missing data due to incomplete or missing clinical records (n = 37/42).

¶Missing data due to incomplete or missing clinical records (n = 192/244).

║Missing data due to incomplete or missing clinical records (n = 13/19).

††Missing data due to incomplete or missing clinical records (n = 17/19).

### Cost per episode – societal perspective

The mean total cost from the societal perspective per RSV episode was USD 43 (95% CI = 11–76) for non-severe cases and USD 612 (95% CI = 544–680) for severe cases (**Table 2**). Mean costs for the few children with a life-threatening RSV infection were almost twice the total costs for severe cases at USD 1161 (95% CI = 837–1485). Direct medical costs accounted for the highest proportion of the total costs for both severe (91.9%) and life-threatened cases (86.3%) with RSV infection. Total mean costs of non-RSV-associated LRTIs were much lower for non-severe cases (USD 19 *vs.* USD 43), comparable for severe cases (USD 586 *vs.* USD 612), and 1.5 times higher for life-threatened cases (USD 1755 *vs.* USD 1161). Total costs differed significantly by RSV status (*P* < 0.001) and were higher for RSV-positive (USD 482; 95% CI = 415–549) compared to RSV-negative cases (USD 285; 95% CI = 221–348). Costs were predominantly influenced by overhead expenses and the length of stay. When cases with a length of stay exceeding ten days are excluded, costs for life-threatened cases for RSV-positive as well as non-RSV cases become more comparable (Table S1 in the **Online Supplementary Document**).

**Table 2:** Societal cost (in 2023 USD) per LRTI episode for RSV-positive and RSV-negative children <2 years old, by severity level

	RSV-positive (n = 159)			RSV-negative (n = 385)		
	**Non-severe (n = 42)**	**Severe (n = 111)**	**Life-threatened (n = 6)**	**Non-severe (n = 244)**	**Severe (n = 122)**	**Life-threatened (n = 19)**
**Total costs**						
x̄ (95% CI)	43.42 (11.34–75.50)	611.78 (543.91–679.64)	1160.70 (836.70–1484.70)	19.17 (15.82–22.51)	586.12 (484.92–687.31)	1755.47 (1110.42–2400.51)
MD (IQR)	18.13 (10.63–37.69)	550.03 (417.56–724.99)	1216.59 (1,008.67–1319.18)	13.35 (10.31–19.84)	461.75 (306.18–711.95)	1321.51 (875.95–3086.02)
**Direct medical costs**						
x̄ (95% CI)	13.45 (11.47–15.42)	561.95 (497.51–626.39)	1001.62 (590.03–1413.22)	13.05 (11.22–14.87)	484.49 (402.77–566.22)	1530.94 (960.82–2101.05)
MD (IQR)	10.80 (8.96–16.79)	442.37 (332.01–679.77)	1029.70 (876.94–1255.93)	10.92 (9.04–13.74)	416.29 (286.03–575.41)	1084.76 (269.33–3030.67)
Hospitality/facility-based fees						
*x̄ (95% CI)*	7.47 (7.27–7.68)	524.95 (462.26–587.64)	772.86 (432.84–1112.87)	7.61 (7.11–8.12)	444.39 (365.36–523.42)	1339.49 (820.43–1858.55)
*MD (IQR)*	7.32 (7.32–7.32)	396.78 (264.00–652.16)	750.25 (652.16–1046.59)	7.32 (7.32–7.32)	391.30 (260.87–521.75)	782.67 (260.87–2608.66)
Medication costs						
*x̄ (95% CI)*	5.03 (3.65–6.41)	7.70 (5.85–9.56)	65.32 (4.92–125.72)	4.69 (4.08–5.30)	10.43 (7.54–13.32)	20.89 (8.29–33.48)
*MD (IQR)*	3.48 (1.64–7.43)	4.70 (1.05–10.74)	37.36 (13.16–75.76)	3.56 (1.72–6.34)	5.28 (1.58–13.83)	9.43 (6.98–27.17)
Laboratory costs						
*x̄ (95% CI)*	0.00 (0.00–0.00)	12.65 (10.37–14.93)	118.75 (28.04–209.45)	1.93 (0.00–5.19)	11.99 (9.83–14.15)	107.12 (62.09–152.15)
*MD (IQR)*	0.00 (0.00–0.00)	6.26 (6.26–12.52)	71.56 (30.69–237.30)	0.00 (0.00–0.00)	6.26 (6.26–10.17)	88.82 (0.00–189.03)
Imaging costs						
*x̄ (95% CI)*	2.09 (0.00–6.06)	14.13 (13.04–15.22)	26.09 (12.29–39.89)	0.00 (0.00–0.00)	14.42 (13.10–15.74)	46.09 (23.63–68.55)
*MD (IQR)*	0.00 (0.00–0.00)	15.65 (15.65–15.65)	23.48 (15.65–46.96)	0.00 (0.00–0.00)	15.65 (15.65–15.65)	39.13 (0.00–78.26)
Miscellaneous costs						
*x̄ (95% CI)*	1.78 (0.00–4.45)	0.83 (0.24–1.41)	11.82 (0.00–25.14)	0.00 (0.00–0.00)	0.15 (0.03–0.27)	11.60 (0.69–22.52)
*MD (IQR)*	0.00 (0.00–0.00)	0.00 (0.00–0.27)	1.07 (0.00–30.60)	0.00 (0.00–0.00)	0.00 (0.00–0.00)	0.53 (0.00–1.34)
Procedure costs						
*x̄ (95% CI)*	1.78 (0.00–3.99)	2.51 (1.22–3.79)	6.78 (0.00–16.30)	1.25 (0.03–2.48)	2.06 (0.87–3.24)	5.01 (0.43–9.59)
*MD (IQR)*	0.00 (0.00–0.16)	0.00 (0.00–2.23)	0.00 (0.00–7.83)	0.00 (0.00–0.00)	0.00 (0.00–0.31)	0.00 (0.00–4.77)
**Direct non-medical costs**						
x̄ (95% CI)*	5.56 (2.90–8.22)	15.68 (11.78–19.57)	25.05 (0.00–62.09)	3.37 (2.29–4.44)	26.05 (18.12–33.98)	10.93 (0.23–21.64)
MD (IQR)	0.71 (0.00–6.42)	5.37 (1.97–22.85)	4.63 (1.57–12.31)	0.47 (0.00–2.82)	12.32 (3.20–28.17)	1.22 (0.47–13.46)
Transport costs						
*x̄ (95% CI)*	0.91 (0.03–1.78)	5.95 (4.20–7.70)	23.98 (0.00–61.44)	0.72 (0.37–1.07)	8.23 (5.09–11.38)	5.04 (1.16–8.92)
*MD (IQR)*	0.47 (0.00–0.94)	2.27 (1.03–4.72)	1.97 (0.47–12.31)	0.00 (0.00–0.47)	3.29 (1.13–8.13)	0.56 (0.00–4.48)
Meal costs						
*x̄ (95% CI)*	0.16 (0.00–0.37)	5.37 (2.74–8.01)	0.00 (0.00–0.00)	0.12 (0.07–0.17)	8.45 (3.33–13.57)	0.05 (0.00–0.14)
*MD (IQR)*	0.00 (0.00–0.00)	0.00 (0.00–2.90)	0.00 (0.00–0.00)	0.00 (0.00–0.00)	0.00 (0.00–0.00)	0.00 (0.00–0.00)
Caretaker costs						
*x̄ (95% CI)*	0.00 (0.00–0.00)	0.57 (0.00–1.38)	0.00 (0.00–0.00)	0.71 (0.00–1.59)	1.18 (0.00–3.46)	0.00 (0.00–0.00)
*MD (IQR)*	0.00 (0.00–0.00)	0.00 (0.00–0.00)	0.00 (0.00–0.00)	0.00 (0.00–0.00)	0.00 (0.00–0.00)	0.00 (0.00–0.00)
**Indirect costs**						
x̄ (95% CI)	24.41 (0.00–56.12)	34.15 (21.84–46.47)	134.03 (18.09–249.98)	2.75 (1.31–4.19)	75.57 (39.54–111.61)	213.60 (26.05–401.14)
MD (IQR)	0.00 (0.00–3.69)	8.44 (0.00–30.08)	80.43 (0.00–264.35)	0.00 (0.00–1.06)	12.67 (0.00–46.96)	14.01 (0.00–227.86)
Lost income						
*x̄ (95% CI)*	3.99 (0.74–7.23)	4.29 (1.16–7.41)	18.26 (0.00–48.54)	0.76 (0.04–1.47)	6.39 (0.00–12.82)	13.02 (0.00–30.38)
*MD (IQR)*	0.00 (0.00–0.00)	0.00 (0.00–0.00)	0.00 (0.00–7.83)	0.00 (0.00–0.00)	0.00 (0.00–0.00)	0.00 (0.00–0.00)
Lost productivity						
*x̄ (95% CI)*	20.15 (0.00–50.87)	23.26 (12.88–33.64)	120.67 (0.00–255.48)	1.60 (0.53–2.67)	61.77 (30.38–93.15)	194.78 (4.35–385.20)
*MD (IQR)*	0.00 (0.00–0.49)	0.00 (0.00–20.81)	0.00 (0.00–253.89)	0.00 (0.00–0.00)	0.00 (0.00–30.58)	0.00 (0.00–175.50)
Lost leisure						
*x̄ (95% CI)*	0.28 (0.00–0.60)	6.60 (5.05–8.16)	15.22 (2.52–27.92)	0.40 (0.20–0.59)	7.92 (5.68–10.16)	16.06 (6.29–25.83)
*MD (IQR)*	0.00 (0.00–0.00)	4.22 (0.00–8.44)	9.76 (0.00–29.56)	0.00 (0.00–0.00)	4.22 (0.00–12.67)	7.39 (0.00–25.33)

CI – confidence interval, IQR – interquartile range, MD – median, RSV – respiratory syncytial virus, LRTI – lower respiratory tract infection, x̄ – mean

*Besides transport, meal, and caretaker costs, lodging costs are also included in this category; however, only a few cases have costs here and are not shown in the table.

### Health system costs

Mean direct medical costs for the health system (**Table 3**) were comparable for outpatient cases who tested positive for RSV and those who tested negative (USD 7.25 *vs.* USD 7.27; *P* = 0.0968), but lower for severe RSV-negative cases (USD 449 *vs.* USD 550; *P* = 0.0068) and higher for non-RSV life-threatened cases (USD 1508 *vs.* USD 928; *P* = 0.5043). As expected, costs increased with longer hospital stays and severity of illness, regardless of RSV status. Additionally, medical expenses for more severe cases included higher costs for medication, as well as laboratory and imaging services.

**Table 3:** Direct medical cost (in 2023 USD) covered by the health system per LRTI episode for RSV-positive and RSV-negative children <2 years old by severity level

	RSV-positive (n = 159)			RSV-negative (n = 385)		
	**Non-severe (n = 42)**	**Severe (n = 111)**	**Life-threatened (n = 6)**	**Non-severe (n = 244)**	**Severe (n = 122)**	**Life-threatened (n = 19)**
**Direct medical costs**						
x̄ (95% CI)*	7.25 (7.24–7.26)	549.91 (485.76–614.06)	928.46 (505.52–1351.39)	7.27 (7.25–7.30)	448.92 (383.19–514.64)	1508.01 (945.27–2070.74)
MD (IQR)	7.24 (7.24–7.24)	435.39 (312.04–674.18)	931.00 (706.52–1212.33)	7.24 (7.24–7.24)	410.72 (280.55–550.13)	1076.47 (264.55–2981.56)
Hospitality/facility-based fees						
*x̄ (95% CI)*	7.25 (7.24–7.25)	521.29 (458.54–584.04)	760.86 (418.08–1103.64)	7.24 (7.24–7.25)	420.98 (356.41–485.55)	1337.99 (818.56–1857.43)
*MD (IQR)*	7.24 (7.24–7.24)	388.17 (260.87–649.03)	717.38 (652.16–1043.46)	7.24 (7.24–7.24)	388.17 (257.74–518.60)	782.60 (257.74–2605.53)
Medication costs						
*x̄ (95% CI)*	0.01 (0.00–0.02)	1.77 (1.32–2.21)	15.77 (4.47–27.07)	0.00 (0.00–0.00)	1.74 (1.38–2.11)	13.63 (1.67–25.59)
*MD (IQR)*	0.00 (0.00–0.00)	0.89 (0.43–2.08)	7.73 (6.81–33.48)	0.00 (0.00–0.00)	0.89 (0.66–2.08)	6.81 (6.22–11.74)
Laboratory costs						
*x̄ (95% CI)*	0.00 (0.00–0.00)	12.56 (10.28–14.85)	113.92 (20.46–207.38)	0.11 (0.00–0.22)	11.95 (9.80–14.11)	107.12 (62.09–152.15)
*MD (IQR)*	0.00 (0.00–0.00)	6.26 (6.26–10.17)	57.08 (30.69–237.30)	0.00 (0.00–0.00)	6.26 (6.26–10.17)	88.82 (0.00–189.03)
Imaging costs						
*x̄ (95% CI)*	2.09 (0.00–6.06)	14.13 (13.04–15.22)	26.09 (12.29–39.89)	0.00 (0.00–0.00)	13.98 (12.79–15.17)	46.09 (23.63–68.55)
*MD (IQR)*	0.00 (0.00–0.00)	15.65 (15.65–15.65)	23.48 (15.65–46.96)	0.00 (0.00–0.00)	15.65 (15.65–15.65)	39.13 (0.00–78.26)
Procedure costs						
*x̄ (95% CI)*	1.41 (0.00–4.10)	0.83 (0.24–1.41)	11.82 (0.00–25.14)	0.00 (0.00–0.00)	0.15 (0.03–0.27)	11.60 (0.69–22.52)
*MD (IQR)*	0.00 (0.00–0.00)	0.00 (0.00–0.27)	1.07 (0.00–30.60)	0.00 (0.00–0.00)	0.00 (0.00–0.00)	0.53 (0.00–1.34)

CI – confidence interval, IQR – interquartile range, MD – median, RSV – respiratory syncytial virus, LRTI – lower respiratory tract infection, x̄ – mean

*Miscellaneous costs are part of direct medical costs; however, those costs that are >95% covered by the households and are not shown.

### Household costs

Of 544 caregivers, 516 reported out-of-pocket costs (**Table 4**). Overall, direct medical out-of-pocket costs accounted for between 28.2% and 36.6% of the total out-of-pocket cost related to one RSV illness episode. The mean direct medical out-of-pocket costs for RSV-positive patients were USD 6 (95% CI = 4–8) for non-severe cases, USD 12 (95% CI = 10–14) for severe cases, and USD 73 (95% CI = 0–170) for life-threatened cases. The overall mean out-of-pocket costs for RSV-positive patients receiving inpatient care (Table S2 in the **Online Supplementary Document**) were almost three times higher compared to outpatient cases (USD 43 *vs.* USD 16). Direct non-medical out-of-pocket costs, such as transportation, food, and caretaker costs, accounted for between 19.0% and 40.6% of the total household costs related to one RSV illness episode. Indirect out-of-pocket costs accounted for approximately 25% of the overall out-of-pocket expenses for RSV-positive cases, irrespective of the severity. Among non-RSV cases, the overall out-of-pocket costs for outpatients were significantly lower than those for RSV cases (USD 10 *vs*. USD 16; *P* = 0.0321). Similarly, we found cost differences for severe and life-threatened cases; however, these were not statistically significant (*P* = 0.1496 and *P* = 0.4004, respectively). Most families of patients with non-severe (48.6%), severe (87.0%), and life-threatening (100.0%) illnesses reported having used savings to cover disease-related treatment costs.

**Table 4:** Household out-of-pocket costs (USD) per LRTI episode for RSV-positive and RSV-negative children <2 years old by severity level (2023)

	RSV-positive (n = 159)			RSV-negative (n = 385)		
	**Non-severe (n = 42)**	**Severe (n = 111)**	**Life-threatened (n = 6)**	**Non-severe (n = 244)**	**Severe (n = 122)**	**Life-threatened (n = 19)**
**Total costs**						
x̄ (95% CI)	16.02 (11.33–20.71)	38.61 (31.70–45.52)	131.69 (14.84–248.53)	10.30 (7.48–13.11)	75.94 (30.26–121.61)	62.94 (17.40–108.48)
MD (IQR)	8.10 (2.90–26.76)	28.58 (14.41–44.99)	60.62 (15.42–294.66)	5.46 (2.78–11.00)	33.03 (15.65–66.33)	25.64 (6.42–65.66)
**Direct medical costs**						
x̄ (95% CI)*	6.19 (4.21–8.17)	12.04 (9.88–14.20)	73.16 (0.00–169.99)	5.78 (3.95–7.60)	35.58 (0.00–78.56)	22.93 (0.00–45.91)
MD (IQR)	3.56 (1.72–9.55)	8.22 (4.71–14.09)	20.43 (10.25–43.60)	3.68 (1.80–6.50)	9.18 (4.56–18.79)	6.35 (3.14–18.87)
Hospitality/facility-based fees						
*x̄ (95% CI)*	0.23 (0.02–0.44)	3.66 (3.36–3.96)	12.00 (0.00–31.04)	0.37 (0.00–0.88)	23.41 (0.00–64.21)	1.49 (0.73–2.26)
*MD (IQR)*	0.08 (0.08–0.08)	3.13 (3.13–3.21)	1.57 (0.00–3.13)	0.08 (0.08–0.08)	3.13 (3.13–5.48)	0.08 (0.00–3.13)
Medication costs						
*x̄ (95% CI)*	5.02 (3.64–6.41)	5.94 (4.20–7.67)	49.55 (0.00–110.68)	4.69 (4.08–5.30)	8.69 (5.85–11.53)	7.26 (2.65–11.86)
*MD (IQR)*	3.48 (1.64–7.43)	3.14 (0.08–7.83)	16.90 (6.35–38.12)	3.56 (1.72–6.34)	3.47 (0.08–10.25)	1.77 (0.17–12.76)
Laboratory costs						
*x̄ (95% CI)*	0.00 (0.00–0.00)	0.08 (0.00–0.17)	4.83 (0.00–12.56)	0.42 (0.00–1.17)	0.04 (0.00–0.09)	0.00 (0.00–0.00)
*MD (IQR)*	0.00 (0.00–0.00)	0.00 (0.00–0.00)	0.00 (0.00–2.35)	0.00 (0.00–0.00)	0.00 (0.00–0.00)	0.00 (0.00–0.00)
Miscellaneous costs						
*x̄ (95% CI)*	1.87 (0.00–4.16)	2.43 (1.14–3.71)	6.78 (0.00–16.30)	1.17 (0.03–2.32)	1.95 (0.76–3.13)	4.75 (0.52–8.98)
*MD (IQR)*	0.00 (0.00–0.16)	0.00 (0.00–1.80)	0.00 (0.00–7.83)	0.00 (0.00–0.00)	0.00 (0.00–0.00)	0.00 (0.00–4.77)
**Direct non-medical costs**						
x̄ (95% CI)†	5.56 (2.90–8.22)	15.68 (11.78–19.57)	25.05 (0.00–62.09)	3.37 (2.29–4.44)	26.05 (18.12–33.98)	10.93 (0.23–21.64)
MD (IQR)	0.71 (0.00–6.42)	5.37 (1.97–22.85)	4.63 (1.57–12.31)	0.47 (0.00–2.82)	12.32 (3.20–28.17)	1.22 (0.47–13.46)
Transport costs						
*x̄ (95% CI)*	0.91 (0.03–1.78)	5.95 (4.20–7.70)	23.98 (0.00–61.44)	0.72 (0.37–1.07)	8.23 (5.09–11.38)	5.04 (1.16–8.92)
*MD (IQR)*	0.47 (0.00–0.94)	2.27 (1.03–4.72)	1.97 (0.47–12.31)	0.00 (0.00–0.47)	3.29 (1.13–8.13)	0.56 (0.00–4.48)
Meal costs						
*x̄ (95% CI)*	0.16 (0.00–0.37)	5.37 (2.74–8.01)	0.00 (0.00–0.00)	0.12 (0.07–0.17)	8.45 (3.33–13.57)	0.05 (0.00–0.14)
*MD (IQR)*	0.00 (0.00–0.00)	0.00 (0.00–2.90)	0.00 (0.00–0.00)	0.00 (0.00–0.00)	0.00 (0.00–0.00)	0.00 (0.00–0.00)
**Indirect costs**						
x̄ (95% CI)	4.26 (0.85–7.68)	10.89 (7.16–14.62)	33.48 (0.00–74.07)	1.15 (0.42–1.88)	14.31 (7.38–21.24)	29.07 (4.52–53.63)
MD (IQR)	0.00 (0.00–0.00)	4.22 (0.00–14.09)	13.68 (0.00–29.56)	0.00 (0.00–0.00)	4.22 (0.00–12.67)	12.67 (0.00–27.45)
Lost income						
*x̄ (95% CI)*	3.99 (0.74–7.23)	4.29 (1.16–7.41)	18.26 (0.00–48.54)	0.76 (0.04–1.47)	6.39 (0.00–12.82)	13.02 (0.00–30.38)
*MD (IQR)*	0.00 (0.00–0.00)	0.00 (0.00–0.00)	0.00 (0.00–7.83)	0.00 (0.00–0.00)	0.00 (0.00–0.00)	0.00 (0.00–0.00)
Lost leisure						
*x̄ (95% CI)*	0.28 (0.00–0.60)	6.60 (5.05–8.16)	15.22 (2.52–27.92)	0.40 (0.20–0.59)	7.92 (5.68–10.16)	16.06 (6.29–25.83)
*MD (IQR)*	0.00 (0.00–0.00)	4.22 (0.00–8.44)	9.76 (0.00–29.56)	0.00 (0.00–0.00)	4.22 (0.00–12.67)	7.39 (0.00–25.33)

CI – confidence interval, IQR – interquartile range, MD – median, RSV – respiratory syncytial virus, LRTI – lower respiratory tract infection, x̄ – mean

*Direct medical out-of-pocket costs for imaging and other services are negligible and are not shown here.

†Besides transport and meal costs, caretaker and lodging costs are included in this category; however, only a few cases have costs here and are not shown in the table.

## DISCUSSION

This is the first prospective study to evaluate costs related to LRTI throughout an entire RSV season in Mozambique, reporting direct medical, direct non-medical, and indirect costs from health system and household perspectives. It provides a further understanding of RSV’s impact by estimating the economic burden of RSV-related facility visits in a low-income country. This information is crucial for informing policy decision-makers and evaluating new intervention strategies to prevent RSV infections. It provides insight into the direct medical, non-medical, and indirect costs incurred due to RSV in Maputo, Mozambique. RSV-related hospitalisations in children <2 years old caused a substantial economic burden on the Mozambican health system and households. Inpatient care costs for children treated at the ICU were approximately twice as high as those for patients treated in the paediatric wards.

In our introduction, we highlighted the lack of data on RSV-related health burden and costs in Mozambique. We referenced a hospital-based study conducted in Maputo, which reported an incidence rate of RSV infections of 23.1% among children <2 years old [8]. Moreover, a recent investigation on intensive care in Maputo found that 35.1% of patients <2 years old admitted with extended severe acute respiratory infection symptoms tested positive for RSV [9]. Our study shows once again the high burden of RSV on the health system in Mozambique, with 29.2% of the included patients testing positive for RSV.

Globally, RSV-related cost data from LMICs are sparse. Malawi is the only low-income country in Africa with available cost-of-illness data for RSV. Mean costs per RSV episode reported in Malawi in 2018 were USD 62 for inpatient cases and USD 13 for outpatients [14]. Taking inflation into account, RSV-related costs in Malawi corresponded to 11.2% and 2.3% of the GDP per capita for inpatients and outpatients, respectively, in 2023. The costs from our study in Mozambique are much higher, contributing to 105.2% for inpatient cases and 7.1% of GDP per capita for outpatient cases. Another cost-of-illness study from neighbouring upper-middle-income South Africa demonstrated comparable costs for hospitalised children, with the cost per severe RSV illness episode totalling USD 681 in 2016 (USD 865 when adjusted for inflation to 2023) [15]. This suggests that, despite the economic differences between Mozambique and South Africa, healthcare infrastructure, treatment practices, and cost drivers may be more aligned than expected.

Several factors could explain the observed differences across countries, including variations in the availability and accessibility of healthcare. While Mozambique and Malawi are both classified as low-income countries, the quality of care may differ. In some cases, the quality of care in these countries may be closer to that observed in South African facilities, despite the differences in economic status. Additionally, differences in treatment protocols and clinical guidelines can significantly impact illness-related costs and contribute to cost discrepancies. Moreover, the completeness of the costs included in our study might also contribute to the differences observed when compared to other studies. These inter-country differences highlight the need of country-specific cost estimates to inform decision-making at the national level.

A similar study conducted in Mopeia, a more rural area of Mozambique, estimated the costs of malaria in children [16]. Although most costs were incurred by younger children, it is important to note that this study focussed on costs incurred by children under five years old. The mean costs for the society per severe episode of malaria were reported as USD 108 (USD 131 when adjusted for inflation to 2023). While the treatment and management of malaria and respiratory illness may not be directly comparable, the costs estimated for severe RSV-positive cases from our study contribute to the findings by highlighting the substantial economic impact of infectious diseases within the Mozambican health system. Costs for malaria care in Mopeia may be lower due to the rural setting and the nature of district hospitals, where overhead costs are likely lower and the level of care provided may be less comprehensive, resulting in lower overall costs.

Several studies have identified catastrophic health expenditures related to severe RSV illness from 24% up to 100% of the monthly income [14]. In Malawi, out-of-pocket costs accounted for 24% of the monthly household income. In South Africa, severe RSV illness was associated with overwhelming out-of-pocket personal expenses averaging more than 100% of the mean monthly income. A similar observation was made in Kenya, another LMIC where over 90% of the households reported adverse financial impacts due to the costs of RSV illness. While the Government of Mozambique strives to provide coverage for direct medical costs for patients, families still face substantial out-of-pocket expenses. For severe and life-threatened RSV cases, these costs remain high, ranging from approximately 43% to 145% of the monthly minimum wage per person (USD 91 in 2023). In our study, all families with children admitted to the ICU reported having to use their savings to cover expenses. Addressing this economic burden is crucial to reducing financial strain on families, lowering childhood mortality, and advancing poverty reduction efforts.

Given the median age of 3.3 months of children with life-threatening RSV infection in our study, both mAbs and maternal vaccination would likely have a high impact on preventing paediatric life-threatening RSV infection in Mozambique. Our detailed quantification of RSV-related costs is key for decision-making, prioritising future preventive strategies, and achieving effective allocation of health resources. Moreover, data could be used for estimating the full impact of introducing a mAb or maternal vaccine in Mozambique. The data derived here provide a basis for subsequent cost-effectiveness analyses, allowing for the economic impact of mAbs and maternal vaccines on RSV to be better evaluated and inform decisions regarding the most cost-effective intervention strategy to be introduced in Mozambique.

This study had several limitations. First, a major weakness is the lack of accessible cost data from the health system. Unit expenses for each category were obtained from the administrative office due to a lack of individual documentation in the public domain and are sometimes based on charges rather than actual costs to the health system. Additionally, by relying on administrative cost data, it is possible that not all relevant costs were captured, which could lead to inaccuracies in the cost estimates. Second, we only captured incurred costs without considering the quality of care received. Third, the study population consisted of patients from a single referral hospital and one healthcare centre in the capital of Mozambique, limiting the generalisability of the findings to the whole country. Fourth, a high participant/caregiver burden (due to lengthy interviews) potentially affected the quality of data and reduced participation rates. Furthermore, follow-up interviews conducted via phone calls may have contributed to lower response rates when compared to regular in-person follow-up visits. We note, however, that study adherence was high. Sixth, the validity of the household cost findings may be compromised by reliance on self-reported cost data from parents/caregivers, since discussing costs and income can be sensitive in Mozambique. Lastly, using the average wage as a proxy may not fully capture individual income differences, potentially introducing bias into the estimates of indirect costs and therefore the overall household costs.

## CONCLUSIONS

Our study highlights the substantial financial burden that (severe) RSV imposes not only on the government but also on the households. This economic strain further underscores the urgent need for preventive strategies to mitigate long-term healthcare costs and reduce the economic impact on families. The findings reinforce the importance of ensuring that effective and affordable interventions are accessible, particularly in LMICs, as Mozambique where the burden of RSV is disproportionally high. It is crucial for stakeholders to fully understand the economic impact of RSV to evaluate the potential implementation of immunisation strategies. Prioritising prevention strategies and ensuring access to effective interventions for all children in Mozambique should be a key focus. We call on stakeholders to act by assessing the cost-effectiveness of available interventions and working towards making RSV prevention in Mozambique a reality.

## Additional Material


Online Supplementary Document
