# Supplementary material for: Estimating the economic burden of respiratory syncytial virus infection among children <2 years old receiving care in Maputo, Mozambique
Source: J Glob Health. 2025 Apr 11;15:04076. doi: 10.7189/jogh.15.04076 (PMC11986275; doi:10.7189/jogh.15.04076)
Supplement: Online Supplementary Document [file jogh-15-04076-s001.pdf]

Supplementary material to:
Rave N, Mussá T, Nguyen A, Pecenka C, Shaaban FL, Bont LJ; RSV GOLD III – Health Economics Study Group. Estimating the economic burden of respiratory syncytial virus infection among children < 2 years old receiving care in Maputo, Mozambique. J Glob Health. 2025;15:04076.

With the RSV GOLD III – Health Economics Study we evaluate costs associated with acute lower respiratory tract infections (LRTIs) in children <2 years old in four developing countries (Ghana, Mozambique, Nepal, and Nigeria), during one local respiratory season. Here we provide details on the country-specific study design conducted in Mozambique.

Data collection process

Data were collected prospectively during one local respiratory season, from February until August 2023. Trained study staff administered questionnaires designed for the RSV GOLD III – Health Economics Study, which were translated to Portuguese. Caregiver questions covered demographics and socioeconomic status, pre-facility visit illness history, and related costs. In addition, all costs from the day of visit were reported. Patient medical records were reviewed upon discharge for detailed information on laboratory tests, imaging, procedures, and medication. For both inpatients and outpatients, follow-up phone calls occurred two to four weeks after facility visit/discharge to obtain information on subsequent illness-related costs, whether any additional facility visits took place for illness-related symptoms, and, if so, which costs were incurred. A detailed overview of the study setup can be found in Figure S1. The local study team entered all data into the Castor electronic data capture system [1].

We considered costs from the health system and household perspectives, which we combined to obtain costs from the societal perspective. From the health system perspective, costs included direct medical costs for patient care that were covered by the government, insurers, donors, and international support. Costs from the household perspective included direct medical costs paid out-

of-pocket, direct non-medical expenses for the household, and indirect costs for income and leisure loss. The direct medical costs to provide care, whether covered by the health system or households, included costs in the following categories:

- Hospitality/facility based fees: overhead costs; registration fees; consultation fees; hospital-bed fees including ICU, emergency room, and inpatient-bed fees; doctor/nurse fees; other facility fees.
- Costs for services, procedures, and interventions such as oxygen therapy, mechanical ventilation, physiotherapy, breathing exercises, etc.
- Costs for all laboratory tests.
- Costs for all imaging diagnostic tests.
- Costs for medications, including prescription and over-the-counter drugs, homeopathic medicines, and traditional medicines such as herbs.
- Costs for miscellaneous items such as medical consumables (gloves, masks, etc.) and all other costs.

We used the annual average exchange rate for 2023 of USD 1 = 63.89 MZN to convert between costs in Mozambique metical (MZN) and US dollar [2].

Costs for the health system

We obtained overhead costs (including facility expenses and staff costs) from various administrative departments at Maputo Central Hospital and the 1st of May Health Centre. In addition, for direct medical costs covered by the health system, we collected quantity of health care utilization in all cost categories recorded in the hospital records and obtained unit costs per service provided from the administrative office of Maputo Central Hospital and the 1st of May Health Centre. Data on staff costs at the 1st of May

Health Centre were not available. Only personnel numbers could be obtained. We assumed that salaries were comparable to those at the hospital and used these as a proxy. If unit costs for certain items or services were not available, we estimated costs of resources used instead. For example, we estimated the cost for oxygen therapy via nasal cannula by adding up the costs of nasal cannula kits and oxygen use (including average oxygen volume used per day) [3] and multiplying the sum by duration of use and cost for oxygen supply in hospital. For several donated prescription medications, we could not retrieve the drug costs and treated the cost as missing, recognizing that it would underestimate the drug costs. In the case of missing itemized costs, we used estimates from related hospital records wherever possible.

Costs to the household

A standard registration fee of 200 to 350 MZN (USD 3.13 to USD 5.48) and 1 to 5 MZN (USD 0.02 to USD 0.08) was applicable to all individuals visiting Maputo Central Hospital and the 1st of May Health Centre, respectively, in 2023. At the household level, costs consisted of patient-level out-of-pocket expenses, non-medical costs such as transportation to and from the facility, meals, accommodation, caregiver expenses, and indirect costs such as lost income and leisure time incurred through the course of the disease. Costs included are related to pre-facility visit(s) related to the episode of illness, index visit costs, and post-facility visit costs.

Data analysis

We summarised the demographic and clinical characteristics of all patients. Data were partially missing for 35 cases due to loss-to-follow-up status or unavailable clinical files. These included five mortality cases, whose follow-up files were considered as missing. For demographic and clinical characteristics where less than 10% of the observations were missing, missing values were not reported. Any variable missing more than 10% of potential observations was noted accordingly.

We used a bottom-up costing approach to estimate the treatment costs associated with respiratory illness. In general, direct medical and non-medical costs were calculated by multiplying the quantity used by unit cost for all items across all cost categories. Overhead costs per patient were calculated by summing all available facility-related expenses. Since staff costs were only available from the paediatric department, we used the ratio of beds in the paediatric ward to the total number of beds in the hospital to estimate the overhead costs for the paediatric department. We then added staff costs from the paediatric department to this amount. The total was subsequently divided by the number of patients treated per day and multiplied by the length of stay for each patient in our study. Since each patient paid an out-of-pocket registration fee of at least 200 MZN for inpatients and 5 MZN for outpatients, this amount was subtracted from the overhead costs to avoid duplication of costs. Transportation costs for those using their own vehicles were estimated from travel distance and fuel consumption. For indirect costs, besides lost income reported by the households, we calculated lost productivity by multiplying the time absent from work by daily income to reflect costs from the societal perspective. Missing wages for caregivers were imputed with the mean wage of other respondents. We used minimum wage in Mozambique [4] to monetise the value of lost leisure time by caregivers. Total costs were the sum of direct medical costs, direct non-medical costs, and indirect costs. Furthermore, costs were analysed with respect to severity of cases based on location of admission.

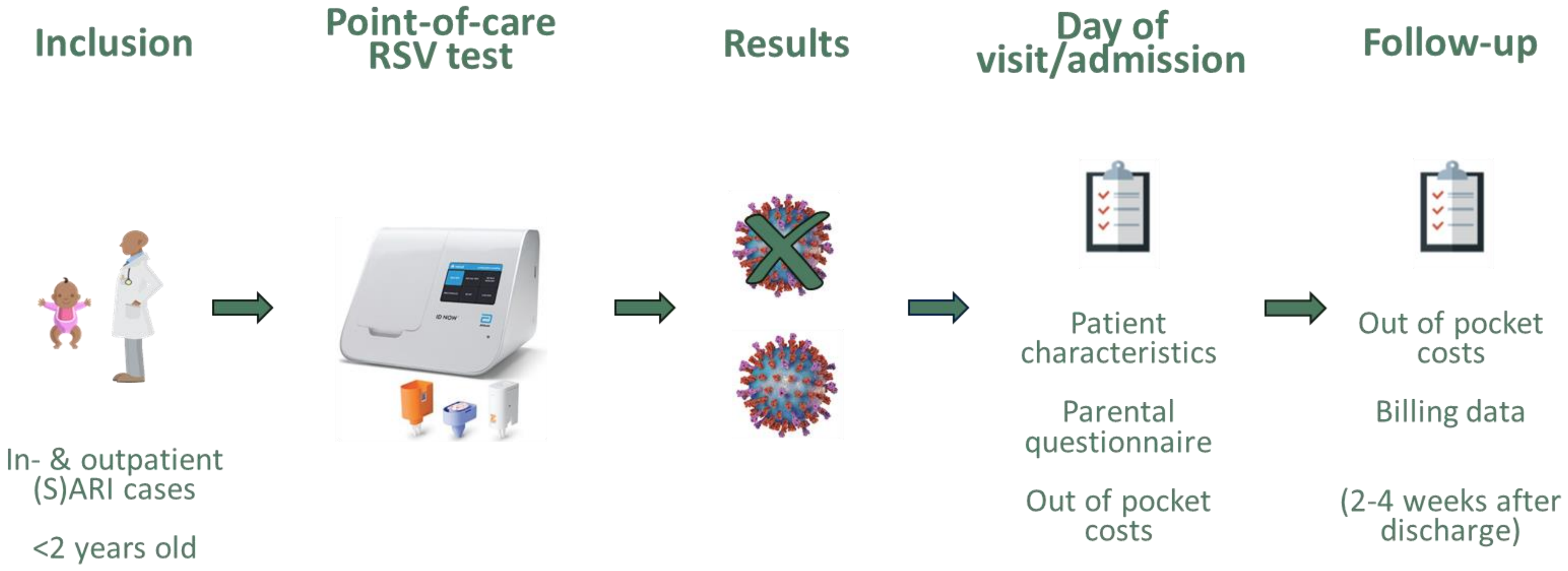

Figure S1. Study setup. RSV – respiratory syncytial virus, (S)ARI – (severe) acute respiratory infection.

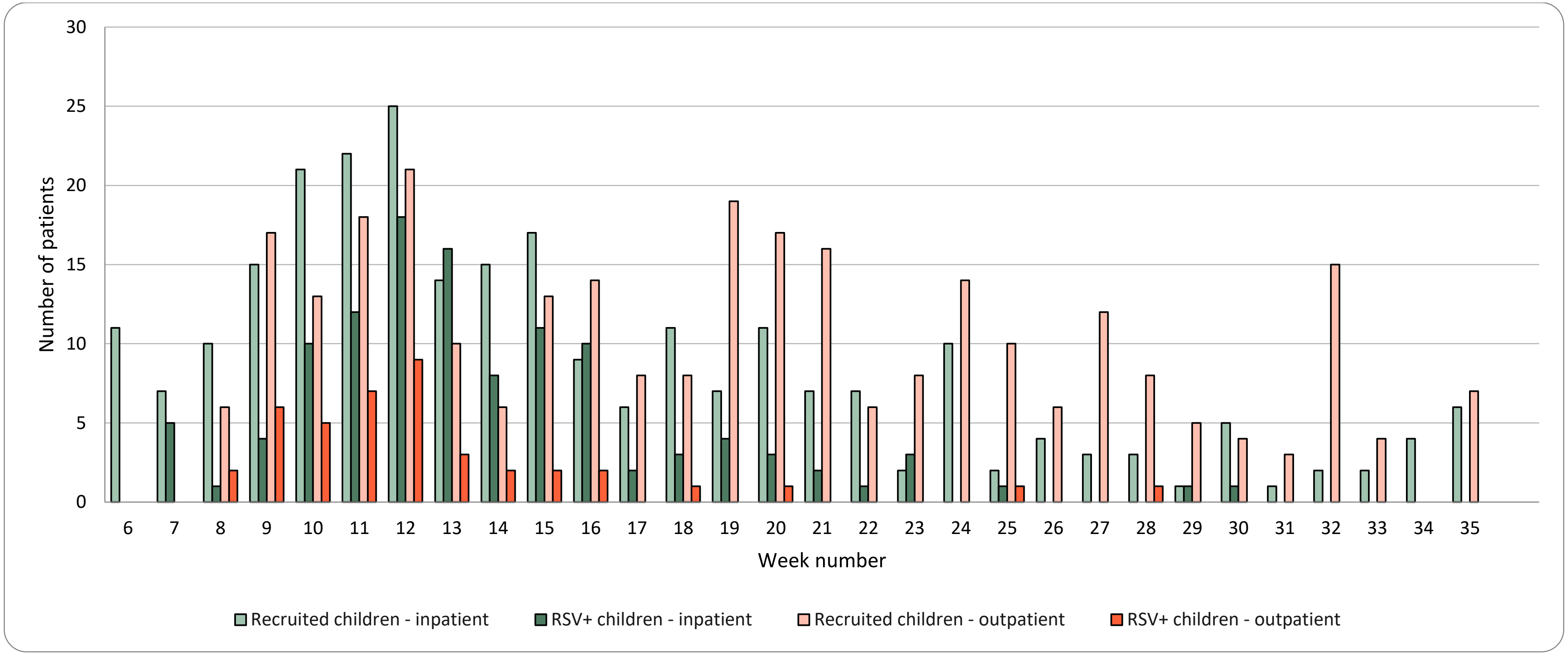

Figure S2. Weekly number of admissions during the study period at the 1st of May Health Centre and Maputo Central Hospital in Mozambique. RSV – respiratory syncytial virus.

Table S1. Overview of costs (in 2023 USD) per lower LRTI episode for RSV-positive and RSV-negative children <2 years old, excluding children with a length of stay exceeding 10 days*, by severity level

	RSV-positive (n=152)			RSV-negative (n=385)		
	**Non-severe (n=42)**	**Severe (n=105)**	**Life-threatened (n=5)**	**Non-severe (n=244)**	**Severe (n=119)**	**Life-threatened (n=10)**
**Societal costs total**						
x̄ (95% CI)	43.42 (11.34-75.05)	550.33 (500.73-599.92)	1033.14 (733.58–1332.70)	19.17 (15.82–22.51)	538.95 (458.63–619.27)	673.44 (396.90–949.99)
MD (IQR)	18.13 (10.63–37.69)	506.14 (416.06–706.57)	1148.04 (1008.67–1285.13)	13.35 (10.31–19.84)	460.41 (305.38–685.86)	901.66 (263.42–1002.68)
Direct medical costs						
X̄ (95% CI)	13.45 (11.47–15.42)	502.09 (455.72–548.46)	843.28 (464.57–1221.98)	13.05 (11.22–14.87)	440.89 (381.55–500.24)	516.99 (242.09–791.88)
MD (IQR)	10.80 (8.96–16.79)	440.15 (324.64–674.22)	1008.67 (876.94–1050.73)	10.92 (9.04–13.75)	414.53 (285.93–570.79)	558.92 (35.09–943.09)
Direct non-medical costs						
X̄ (95% CI)	5.56 (2.90–8.22)	14.51 (10.83–18.19)	29.02 (0.00–71.53)	3.37 (2.29–4.44)	24.09 (18.12–33.98)	5.23 (0.22–21.64)
MD (IQR)	0.71 (0.00–6.42)	4.87 (1.74–21.13)	4.09 (1.57–12.31)	0.47 (0.00–2.82)	11.21 (3.09–27.67)	0.47 (0.00–0.94)
Indirect costs						
X̄ (95% CI)	24.41 (0.00–56.12)	33.73 (22.17–45.28)	160.84 (33.20–288.48)	2.75 (1.31–4.19)	73.97 (36.13–111.81)	151.22 (0.00–380.54)
MD (IQR)	0.00 (0.00–3.69)	8.44 (2.18–30.08)	143.96 (16.89–264.35)	0.00 (0.00–1.06)	12.67 (0.00–43.68)	5.13 (0.00–27.45)
**Health system costs total**						
X̄ (95% CI)	7.25 (7.24–7.26)	490.52 (444.20–536.84)	757.53 (395.60–1119.46)	7.27 (7.26–7.30)	405.26 (365.29–445.23)	505.18 (226.45–783.91)
MD (IQR)	7.24 (7.24–7.24)	418.52 (299.92–671.09)	853.38 (706.52–1008.62)	7.24 (7.24-7.24)	410.42 (280.55–545.08)	555.99 (6.81–938.37)

Table S1. Continued

**Household costs total**						
X̄ (95% CI)	16.02 (11.33–20.71)	37.43 (30.09–44.77)	154.94 (25.27–284.62)	10.30 (7.48–13.11)	72.80 (29.25–116.34)	26.06 (7.37–44.74)
MD (IQR)	8.10 (2.90–26.76)	27.50 (14.41–44.11)	72.80 (48.43–294.66)	5.46 (2.78–11.00)	30.93 (15.49–61.56)	11.22 (5.72–28.51)
Direct medical costs						
X̄ (95% CI)	6.19 (4.21–8.17)	11.57 (9.44–13.70)	85.75 (0.00–206.82)	5.78 (3.95–7.60)	35.63 (0.00–75.70)	11.81 (3.01–20.60)
MD (IQR)	3.56 (1.72–9.55)	7.67 (4.71–13.30)	23.56 (17.31–43.60)	3.68 (1.80–6.50)	8.69 (4.46–18.78)	5.60 (3.38–9.78)
Direct non-medical costs						
X̄ (95% CI)	5.56 (2.90–8.22)	14.51 (10.83–18.19)	29.02 (0.00–71.53)	3.37 (2.29–4.44)	24.09 (16.94–31.24)	5.23 (0.00–11.88)
MD (IQR)	0.71 (0.00–6.42)	4.87 (1.74–21.13)	4.09 (1.57–12.31)	0.47 (0.00–2.82)	11.21 (3.09–27.67)	0.47 (0.00–0.94)
Indirect costs						
X̄ (95% CI)	4.26 (0.85–7.68)	11.35 (7.29–15.42)	40.17 (0.00–85.60)	1.15 (0.42–1.88)	13.08 (5.93–20.22)	9.02 (0.96–17.08)
MD (IQR)	0.00 (0.00–0.00)	4.22 (0.00–14.09)	16.89 (10.46–29.56)	0.00 (0.00–0.00)	4.22 (0.00–12.67)	0.00 (0.00–16.89)

CI – confidence interval, IQR – interquartile range, MD – median, RSV – respiratory syncytial virus, LRTI – lower respiratory tract infection, x̃ – mean.
*Number of patients excluded—For RSV-positive cases: severe, n=6; life-threatened, n=1. For RSV-negative cases: severe, n=3; life-threatened, n=9.

Table S2. Overview of costs (in 2023 USD) per LRTI episode for RSV-positive and RSV-negative children <2 years old, comparing outpatient and inpatient cases

	RSV-positive (n=159)		RSV-negative (n=385)	
	**Outpatient (n=42)**	**Inpatient (n=117)**	**Outpatient (n=244)**	**Inpatient (n=141)**
**Societal costs total**				
X̄ (95% CI)	43.42 (11.34–75.50)	639.93 (570.26–709.59)	19.17 (15.82–22.51)	743.69 (605.95–881.43)
MD (IQR)	18.13 (10.63–37.69)	552.15 (422.29–761.21)	13.35 (10.31–19.84)	486.89 (307.18–826.84)
Direct medical costs				
X̄ (95% CI)	13.45 (11.47–15.42)	584.50 (517.48–651.51)	13.05 (11.22–14.87)	625.50 (505.39–745.61)
MD (IQR)	10.80 (8.96–16.79)	457.51 (409.04–703.56)	10.92 (9.04–13.74)	418.44 (286.03–685.67)
Direct non-medical costs				
X̄ (95% CI)	5.56 (2.90–8.22)	16.16 (11.85–20.46)	3.37 (2.29–4.44)	24.01 (16.99–31.04)
MD (IQR)	0.71 (0.00–6.42)	5.17 (1.97–21.64)	0.47 (0.00–2.82)	10.11 (1.97–24.35)
Indirect costs				
X̄ (95% CI)	24.41 (0.00–56.12)	39.28 (26.32–52.23)	2.75 (1.31–4.19)	94.17 (52.71–135.64)
MD (IQR)	0.00 (0.00–3.69)	8.44 (0.00–31.30)	0.00 (0.00–1.06)	12.67 (0.00–47.90)
**Health system costs total**				
X̄ (95% CI)	7.25 (7.24–7.26)	569.32 (502.94–635.70)	7.27 (7.25–7.30)	591.63 (477.97–705.29)
MD (IQR)	7.24 (7.24–7.24)	445.07 (400.58–676.27)	7.24 (7.24–7.24)	411.12 (280.55–672.11)

Table S2. Continued

**Household costs total**				
X̄ (95% CI)	16.02 (11.33–20.71)	43.38 (33.60–53.17)	10.30 (7.48–13.11)	74.19 (36.57–111.80)
MD (IQR)	8.10 (2.90–26.76)	28.78 (14.41–47.30)	5.46 (2.78–11.00)	30.27 (14.48–65.66)
Direct medical costs				
X̄ (95% CI)	6.19 (4.21–8.17)	15.18 (9.02–21.33)	5.78 (3.95–7.60)	33.87 (0.00–68.69)
MD (IQR)	3.56 (1.72–9.55)	8.45 (5.02–14.37)	3.68 (1.80–6.50)	8.69 (4.15–18.79)
Direct non-medical costs				
X̄ (95% CI)	5.56 (2.90–8.22)	16.16 (11.85–20.46)	3.37 (2.29–4.44)	24.01 (16.99–31.04)
MD (IQR)	0.71 (0.00–6.42)	5.17 (1.97–21.64)	0.47 (0.00–2.82)	10.11 (1.97–24.35)
Indirect costs				
X̄ (95% CI)	4.26 (0.85–7.68)	12.05 (7.84–16.25)	1.15 (0.42–1.88)	16.30 (9.27–23.33)
MD (IQR)	0.00 (0.00–0.00)	4.22 (0.00–15.83)	0.00 (0.00–0.00)	4.22 (0.00–16.89)

CI - confidence interval, IQR - interquartile range, MD - median, RSV - respiratory syncytial virus, LRTI - lower respiratory tract infection, x̃ - mean.

RSV GOLD III – Health Economics Study Group members*

Cameroon:

Frédéric Debellut – Center for Vaccine Innovation and Access, PATH, Geneva, Switzerland
Norbert Fuhngwa – Triangle Research Foundation, Douala, Cameroon
Henshaw Mandi – Triangle Research Foundation, Douala, Cameroon

Ghana:

Rosemary Akuaku – Department of Child Health, Korle Bu Teaching Hospital, Accra, Ghana
Joycelyn Dame – University of Ghana Medical School Korle Bu Teaching Hospital, Accra, Ghana
Amma Ekem – Department of Child Health, Korle Bu Teaching Hospital, Accra, Ghana
Bamenla Goka – University of Ghana Medical School Korle Bu Teaching Hospital, Accra, Ghana
Ebenezer Ntow – Department of Child Health, Korle Bu Teaching Hospital, Accra, Ghana
Kwabena A. Osman – University of Ghana Medical School Korle Bu Teaching Hospital, Accra, Ghana

Mozambique:

Assucênio Chissaque – Instituto Nacional de Saúde, Marracuene district, Maputo, Mozambique; Instituto de Higiene e Medicina Tropical, Universidade Nova de Lisboa, Lisbon, Portugal
Nilsa de Deus – Instituto Nacional de Saúde, Marracuene district, Maputo, Mozambique
Esperança Lourenço Guimarães – Instituto Nacional de Saúde, Marracuene district, Maputo, Mozambique; Instituto de Higiene e Medicina Tropical, Universidade Nova de Lisboa, Lisbon, Portugal
Braiton Maculuve – Ministério da Saúde, Maputo, Mozambique
Elias Manjate – Faculty of Medicine, University Eduardo Mondlane, Maputo
Yara Manjate – Faculty of Medicine, University Eduardo Mondlane, Maputo
Izilda Matimbe – Faculty of Medicine, University Eduardo Mondlane, Maputo
Tufária Mussá – Faculty of Medicine, University Eduardo Mondlane, Maputo
Mirela Pale – Instituto Nacional de Saúde, Marracuene district, Maputo, Mozambique

Cesar Palha – Faculty of Medicine, University Eduardo Mondlane, Maputo
Cristina Sinussene – Faculty of Medicine, University Eduardo Mondlane, Maputo
Farida Zavala – Faculty of Medicine, University Eduardo Mondlane, Maputo

Nepal:
Ram H. Chapagain – Kanti Children's Hospital, Kathmandu, Nepal; Nepal Paediatrics Society, Kathmandu, Nepal
Rita Dhital - Nepal Paediatrics Society, Kathmandu, Nepal
Upendra Dhungana - Public Health Administrator; Ministry of Health and Population. Nepal
Prakash Joshi – Kanti Children's Hospital, Kathmandu, Nepal; Nepal Paediatrics Society, Kathmandu, Nepal
Ranju Karki - Nepal Paediatrics Society, Kathmandu, Nepal
Adita Nepali - Nepal Paediatrics Society, Kathmandu, Nepal
Uttam Paudel - Post Doctorate Researcher (Health Economics), Chulalongkorn University
Arun K. Sharma – Tribhuvan University Teaching Hospital, Institute of Medicine, Kathmandu, Nepal; Nepal Paediatrics Society, Kathmandu, Nepal
Rupesh Shrestha – Tribhuvan University Teaching Hospital, Institute of Medicine, Kathmandu, Nepal
Nirasta Thakili - Nepal Paediatrics Society, Kathmandu, Nepal

Nigeria:
Fadlulai Abdu-Raheem – Department of Paediatrics, Ahmadu Bello University Teaching Hospital, Zaria, Nigeria
Anas Abubakar – Department of Paediatrics, Ahmadu Bello University Teaching Hospital, Zaria, Nigeria
Abdullahi Aminu – Department of Paediatrics, Ahmadu Bello University Teaching Hospital, Zaria, Nigeria
Maria A. Garba – Department of Paediatrics, Ahmadu Bello University Teaching Hospital, Zaria, Nigeria
Fatima J. Giwa – Department of Medical Microbiology, Ahmadu Bello University Teaching Hospital, Zaria, Nigeria
Habiba Lawal – Institute of Child Health, Ahmadu Bello University, Banzazzau, Zaria
Bernsah D. Lawong – Department of Economics, Ahmadu Bello University, Zaria, Nigeria
Abdullahi Musa – Department of Paediatrics, Ahmadu Bello University Teaching Hospital, Zaria, Nigeria
Teddy Naddumba – Center for Vaccine Innovation and Access, PATH, Kampala, Uganda
Aira A. Olorukooba – Department of Paediatrics, Ahmadu Bello University Teaching Hospital, Zaria, Nigeria

Support:
Andrew Clark – Department of Health Services Research and Policy, London School of Hygiene & Tropical Medicine, London, UK
An Nguyen – Center for Vaccine Innovation and Access, PATH, Ho Chi Minh city, Vietnam
Clint Pecenka – Center for Vaccine Innovation and Access, PATH, Seattle, WA, USA

The Netherlands:
Louis J. Bont – University Medical Centre Utrecht, Utrecht, The Netherlands
Neele Rave – University Medical Centre Utrecht, Utrecht, The Netherlands
Farina L. Shaaban – University Medical Centre Utrecht, Utrecht, The Netherlands

* The authors are listed in alphabetical order of their surnames, according to the specific country teams with which they were involved in the study. Team members from University Medical Centre Utrecht, along with supporting staff, were involved at all study sites. A detailed overview of authorship contributions for each country can be found in the respective paper.

References

1 Castor. Castor Electronic Data Capture. 2024. Available: www.castoredc.com. Accessed: 25 April 2024.

2 World Bank Group. Official exchange rate (LCU per USD, period average) 2024. Available:https://data.worldbank.org/indicator/PA.NUS.FCRF?skipRedirection=true&view=map. Accessed: 08 July 2024.

3 Graham H, Bakare AA, Ayede AI, Oyewole OB, Gray A, Peel D, et al. Hypoxaemia in hospitalised children and neonates: A prospective cohort study in Nigerian secondary-level hospitals. EClinicalMedicine. 2019;16:51-63. DOI: 10.1016/j.eclinm.2019.10.009

4 WageIndicator Foundation. Minimum Wages per Country. 2024. Available: https://wageindicator.org/salary/minimum-wage/minimum-wages-per-country. Accessed: 25 April 2024.
